# High Performance Transparent Transistor Memory Devices Using Nano-Floating Gate of Polymer/ZnO Nanocomposites

**DOI:** 10.1038/srep20129

**Published:** 2016-02-01

**Authors:** Chien-Chung Shih, Wen-Ya Lee, Yu-Cheng Chiu, Han-Wen Hsu, Hsuan-Chun Chang, Cheng-Liang Liu, Wen-Chang Chen

**Affiliations:** 1 Department of Chemical Engineering, National Taiwan University, Taipei, 10617 Taiwan; 2 Department of Chemical Engineering and Biotechnology, National Taipei University of Technology, Taipei, 10608; 3 Department of Chemical and Materials Engineering, National Central University, Taoyuan 32001, Taiwan

## Abstract

Nano-floating gate memory devices (NFGM) using metal nanoparticles (NPs) covered with an insulating polymer have been considered as a promising electronic device for the next-generation nonvolatile organic memory applications NPs. However, the transparency of the device with metal NPs is restricted to 60~70% due to the light absorption in the visible region caused by the surface plasmon resonance effects of metal NPs. To address this issue, we demonstrate a novel NFGM using the blends of hole-trapping poly (9-(4-vinylphenyl) carbazole) (PVPK) and electron-trapping ZnO NPs as the charge storage element. The memory devices exhibited a remarkably programmable memory window up to 60 V during the program/erase operations, which was attributed to the trapping/detrapping of charge carriers in ZnO NPs/PVPK composite. Furthermore, the devices showed the long-term retention time (>10^5^ s) and WRER test (>200 cycles), indicating excellent electrical reliability and stability. Additionally, the fabricated transistor memory devices exhibited a relatively high transparency of 90% at the wavelength of 500 nm based on the spray-coated PEDOT:PSS as electrode, suggesting high potential for transparent organic electronic memory devices.

Recently, organic field-effect transistor (OFET) type memory devices have been regarded as a potential candidate for next-generation memory devices because of their advantages of high portability and flexibility, easily integrating structure, non-destructive read-out characteristics and single device structure^1^,^2^,^3^,^4^,^5^,^6^,^7^,^8^. Different from the conventional OFET^9^,^10^, the OFET memory incorporates a distinct charge-storage layer for electrical programmable function, such as ferroelectric materials^11^,^12^, nano-floating gate dielectric or polymer-based electret^1^,^6^,^13^,^14^,^15^,^16^,^17^. Among them, the nano- floating gate architecture have been considered as a promise path to obtain high performance memory devices^2^,^4^,^18^,^19^,^20^,^21^,^22^, because the spatially discrete floating-gate elements effectively act as a charge trapping site^5^,^18^.

In addition to excellent charge trapping capability, optical transparency is another desirable feature for OFET memory^23^,^24^,^25^,^26^. In general, electronic devices with optical transparency provide the advantages for transparent electronics, such as smart glass electronic devices and wearable displays. An embeddable transparent nonvolatile memory devices is strongly demanded to realize highly transparent integrated systems requiring information storage. Previously, Kim and his coworkers^5^ embedded Au NPs (NPs) into a polymer dielectric as a charge storage layer for transparent NFGM devices. However, the Au NPs possessed a strong absorption in the visible region due to their strong surface plasmon resonance effects^2^,^4^,^20^,^21^,^27^. The devices with the Au NPs showed the transparency of 67%, which was much lower than that of the devices without the Au NPs (86%). To improve the transparency, Jang *et al.* reported NFGM devices using single-layer graphene as active channel, and thermal-deposited ultra-thin discontinuous Au layer (6.7 nm) as a charge trapping layer to obtain high transparency transistor memory (up to 80%)^23^. Nevertheless, the highly energy-consumed process, e.g. thermal evaporation process, need to be avoided for environmental issue^13^,^28^,^29^. Alternatively, solution-processed transparent charge trapping layers have the advantage of low-cost and large-area fabrication. Recently, semiconducting nanoscale materials such conjugated polymer nanoparticles^28^ or cobalt ferrite nanoparticles^16^ made from solution process have been used as nano-floating gate. The devices performance are comparable to that of Au NPs, which shows the potential of semiconductor as charge trapping sites.

Zinc oxide have been considered as one of the leading candidates for electronics^30^ because it offers inexpensive synthesis, high crystallinity, transparency and a wide direct band gap (−3.4 eV). ZnO possesses a high transparency over 95% in the visible region, beneficial to the development of transparent electronics. Moreover, its low-lying lowest unoccupied molecular orbital (LUMO) energy level tends to be a strong electron acceptor. ZnO NPs have been employed for organic memory applications. Hirschmann *et al.* employed the ZnO NPs modified by ligand as a charge storage layer in OFET memory^31^. Nevertheless, the memory device showed a poor electrical isolation capability and sustain for only a few minutes. Although ZnO NPs have potential as a charge storage layer for memory applications, the charge retention characteristics need to be further improved.

Herein, we blend the hole-trapping polymer, poly (9-(4-vinylphenyl) carbazole) (PVPK), with transparent ZnO NPs for the NFGM devices to improve their memory performance. The ZnO NPs were synthesized from low-cost solution-based hydrothermal method and modified with n-butylamine, which was favour for the dispersion of the NPs in mixing solvents (chloroform/methanol). PVPK is highly transparent in visible region and has a rigid carbazole moiety which could play a role of a hole-trapping site. Hole/electron storage behavior could be realized through the ZnO-polymer nanocomposite. Additionally, the data retention ability in our system could be significantly enhanced by the method of embedded nanoparticles in insulated polymer layer. For comparison, we also prepared ZnO NP-based memory devices using a common insulating polymer, polystyrene (PS). Precise control and optimization of the ZnO loading amount could tune the memory window and maximize the On/Off current ratios between the programmed and erased states at *V*_g_ = 0 V. The experimental results suggested that the fabricated memory devices with a relatively high transparency, large memory window, high on/off current ratio,, and reliable long term stability for organic nonvolatile memory device applications.

## Results and Discussion

### Preparation of ZnO NPs and characterizations

**Figure 1:**
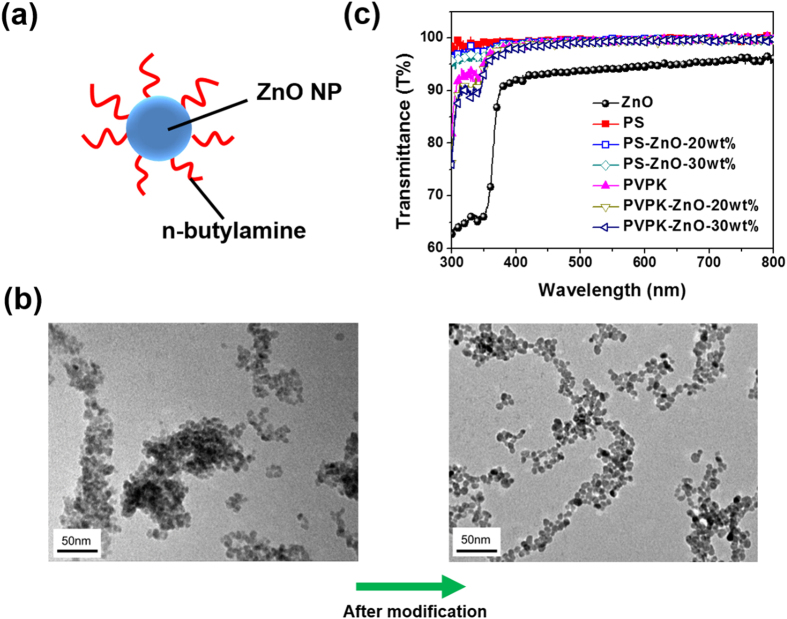
(**a**) Schematic illustration of ZnO NPs modified with the ligands. (**b**) TEM images of the ZnO NPs in PVPK without ligand and after ligand modification (**c**) The transmittance of the charge storage layer with PS and PVPK, and their blends with different ZnO amounts.

ZnO NPs were synthesized by hydrolysis and condensation of zinc acetate dihydrate by potassium hydroxide in methanol using a Zn^2+^: OH^−^ ratio of 1:1.7 and n-butylamine as ligand (Fig. 1a)^32^. The ZnO NPs were dispersed in the polymer matrix using the mixing solvent system of chloroform and methanol at the ratio of 3:1^33^. This process could prevent opaque thin film formation and lead to high transparency^34^. In X-ray diffraction pattern (See supporting information Figure S1), the peaks at 2θ = 31.72, 34.36, 36.18,47.44, and 56.5° are corresponding to the lattice planes (100), (002), (101), (102), and (110) of the hexagonal phase of ZnO^35^, respectively. This diffraction pattern indicates that the prepared ZnO NPs is the wurtzite crystal phase.

Sun *et al.* found that ZnO-PMMA composites without any ligand modification showed severe aggregation of ZnO NPs^36^. The ZnO NPs without being modified with the ligand show a quasi-spherical morphology and are severely aggregated with a diameter of 5.0–10.0 nm in PVPK matrix (Fig. 1b left). After modification with n-butylamine for ZnO NPs, the aggregation in the polymer matrix was suppressed (Fig. 1b right), indicating the significant impact of the ligand on the dispersion of ZnO nanocrystals. The less aggregation in the ligand-modified ZnO NPs may be attributed to the enhanced hydrophobicity of the nanocrystals, which improves the compatibility between the ZnO NPs and the polymer matrix^34^. Thus, the addition of n-butylamine to the nanocrystal solutions is critical to improve the optical properties Furthermore, the mixing solvent (chloroform/methanol) allows the nanocrystals to be dissolved in solution and ensuring the mixed solution of ZnO nanocrystals and PVPK to be homogenous, leading to the uniform composite films

Figure 1c shows the optical transmittance of the ZnO, PS, 20 wt%, 30 wt% ZnO/PS, PVPK, 20 wt% and 30 wt% ZnO/PVPK film. The film thickness was around 30–40 nm for each polymer nanocomposite. As shown in Fig. 1c, the samples showed high transparency from 380 to 780 nm, indicating that the ZnO nanoparticle composition does not affect the optical transmittance of the nanocomposite. This may be attributed to the well-dispersed NPs and less aggregation of the ZnO NPs. Note that the transmittance of spin-coated ZnO thin film is lower compared to those of others, which could be due to the light scattering of high-density NPs aggregated on the substrate surface.

To deeply understand the influence of the ZnO NPs and PVPK, we first studied the device performance and memory behavior of ZnO embedded in polystyrene (PS) matrix. PS is a common insulating dielectric materials without any specific functional groups for charge trapping, which is good polymer matrix to understand the memory behavior of ZnO NPs. Sequentially, the device behavior of the ZnO NPs/PVPK blends were investigated. The detailed results and discussion are shown in the following paragraphs.

### Memory Devices Using ZnO/PS composites as Charge-Storage Layer

**Figure 2:**
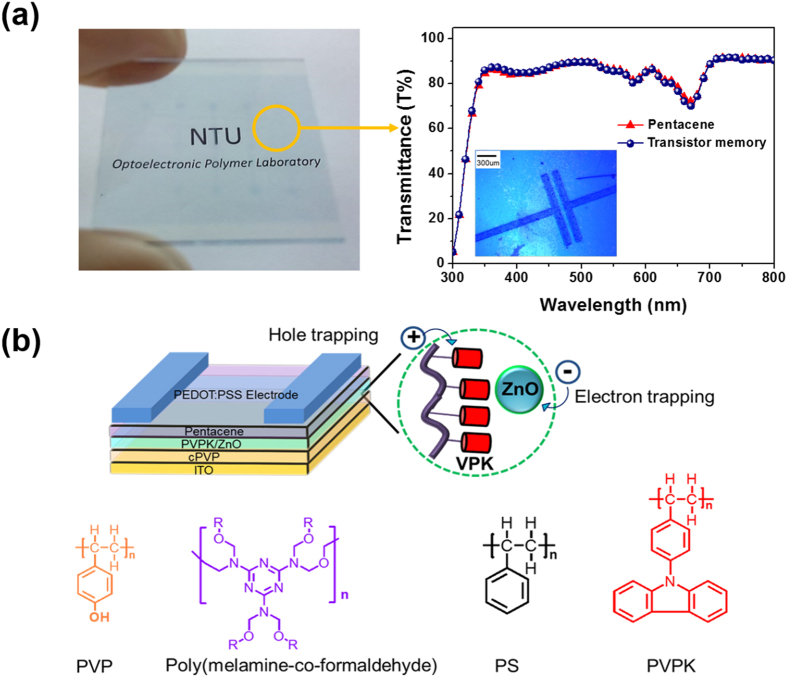
(**a**) Photograph of the transparent OFET memory devices using ZnO/PVPK as charge storage layer and its overall transmittance (**b**) Schematic configuration of the proposed bottom gate/top-contact transistor memory device and the chemical structure of the used polymers.

Figure 2a,b show an image and schematic illustration of the device structures, respectively. To prepare a transparent transistor, a thin-thickness (ca. 30 nm) pentacene crosslinked poly (4-vinylphenol) (cPVP) were used as a charge-transport layer and a dielectric layer, respectively. Poly(3,4-ethylenethiophene)-poly(styrenesulfonate) (PEDOT:PSS) transparent electrode was prepared by spray coating the solution through the shadow mask^37^. Figure 2a shows the image of the fabricated transparent memory device, which is as transparent as an ITO glass. The transmittance for the device at the wavelength of 500 nm is 90%, and the average transparency of 85% over the range from 400 to 800 nm. The device shows a reduced transmittance (around 70%) at the wavelength of 670 nm, which is mainly contributed from the absorption of 30-nm-thick pentacene. This transmittance can be significantly improved when reducing the thickness of the pentacene film. Additionally, the surface morphology of these polymer nanocomposites were investigated using AFM techniques. The AFM images of the electret suggest that the composite film of ZnO/PS or ZnO/PVPK has a relatively smooth surface with the roughness below 2 nm (See supporting information Figure S2).

**Figure 3:**
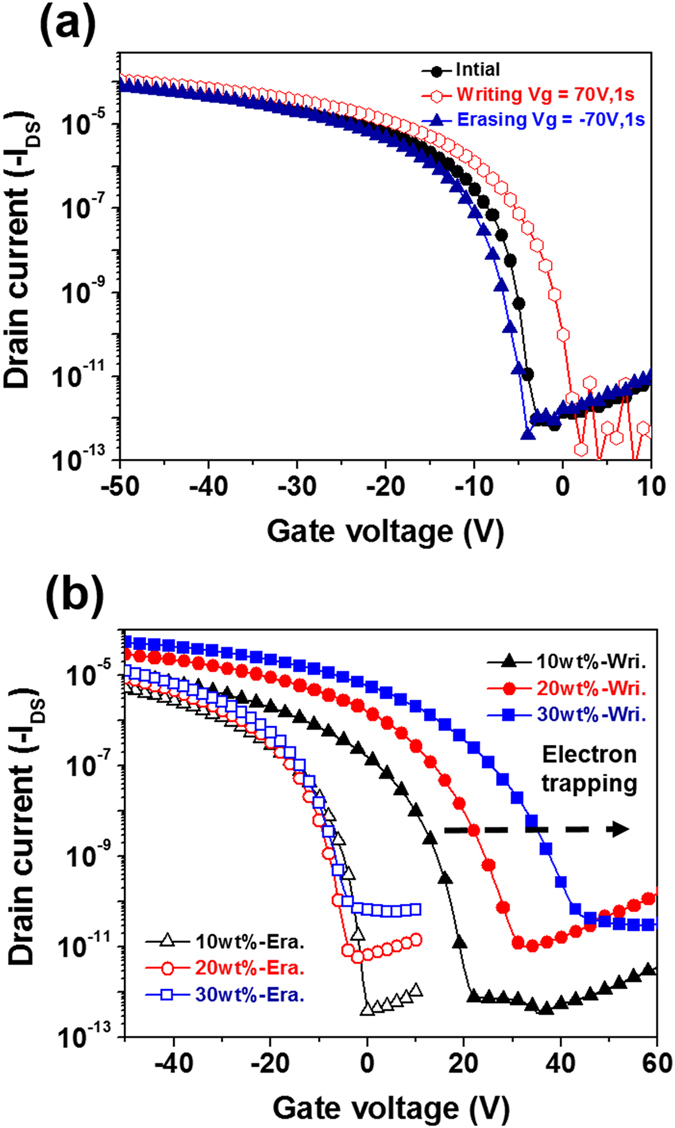
Transfer curves of the fabricated transistor memory devices with (**a**) PS and (**b**) the overlapped curves for the writing and erasing processes with ZnOPS10, ZnOPS20 and ZnOPS30. The drain current was measured at *V*_ds_ = −50 V.

The transfer characteristics of the OFET memory devices using the ZnO/PS composite are shown in Fig. 3, and the detailed transfer characteristics are summarized in Table 1. The transfer curves exhibit a typical p-type accumulation mode with a good current modulation. The field-effect mobility (μ) values of the devices in the saturation region using the film with different ZnO composition (0, 10, 20 and 30 wt%), denoted as PS, ZnOPS10, ZnOPS20, ZnOPS30 are 0.32, 0.36, 0.58, and 0.62 cm^2^ V^−1^ s^−1^, respectively. The corresponding threshold voltages (*V*_th_) are in the range from −9 V to −10 V and the *I*_on_/*I*_off_ current ratios range from 10^5^ to 10^6^, respectively. Intriguingly, it has been observed that the mobility values are enhanced with the increased ZnO amounts. However, there is no significant change from the surface structure of pentacene from AFM (Figure S3), indicating the grain sizes and morphology of pentacene do not dominate the mobility enhancement. Therefore, we believe that the enhanced hole mobility may be attributed to the n-type ZnO nanoparticles blended in the electret, leading to an increased carrier density fulfilling defects in the channel. Similar mobility enhancement have been observed in our previous PCBM nanocomposite system7. Additionally, the off current is also increased with the increment of the ZnO loading. This may indicate the small amounts ZnO at interface causing the doping effects on pentacene layer^1^,^7^. The *I*_on_/*I*_off_ current ratio is up to 10^6^ indicating their potential applications as high performance transistor memory devices. The transistor memories are extended from the regular transistor and operated by the appropriate pulse of the gates voltage. Based on the shifting of transfer curves, high- and low-conductance states at *V*_*g*_ = 0 V can be switched and denoted as two different output signals (0 and 1). Herein, when programming with a positive *V*_g_ pulse (+70 V for 1 s), the onset of the transfer curve shifts to a more positive region and leads to a high-conductance state (ON state) at zero gate voltage (*V*_g_ = 0 V), which serves as a “writing” process. In contrast, once a reverse bias is applied (−70 V for 1 s), the transfer curve moves to the negative direction and results in a low-conductance state (OFF state) at *V*g = 0 V, serving as the “erasing” process. Figure 3b shows the positive and negative shifts of the transfer curves (where *V*_ds_ = −50 V) for the OFET memory devices prepared from different ZnO blending amounts. When we apply the writing process, the entire transfer curves using ZnOPS10, ZnOPS20 and ZnOPS30 as the charge storage layer shift to the positive direction with threshold voltages of 12.27, 17.54 and 21.99 V, respectively, leading to the high drain current (ON state) at *V*_g_ = 0 V. In the erasing process, the transfer curves substantially move to the negative direction for ZnOPS10, ZnOPS20 and ZnOPS30 with threshold voltages of −10.87, −10.21 and −9.27 V respectively. The drain current is dramatically reduced to 6.54 × 10^−12^ A at *V*_g_ = 0 V in the OFF state. Due to no obvious *V*_th_ shifting for pure PS dielectric was observed, the memory windows of the nanocomposite devices are mainly contributed from ZnO NPs. The large *V*_th_ shift in the positive direction strongly suggests the increase of stored charges with enhancing the ZnO composition. The above effect can be attributed to the transfer of charge carrier from semiconductor into the dielectric, which is controlled by the applied gate voltage and known as a tunneling process. The *V*_th_ of the erasing curves is very close for different composition of ZnO NPs, indicating that the ZnO amount does not affect hole trapping. Therefore, we conclude that the trapped electrons are mainly controlled in the ZnO NPs in the forward direction and then detrapped in the backward direction. The memory window (∆*V*_th_) is increased with the increments of ZnO NPs, memory window for ZnOPS10, ZnOPS20and ZnOPS30 are 23.14, 27.75 and 31.26 V respectively. Note that the memory window is denoted as the *V*_th_ difference between the transfer curves through applying a writing and an erasing gate bias.

### Memory Devices Using the ZnO/PVPK composite as Charge-Storage Layer

**Figure 4:**
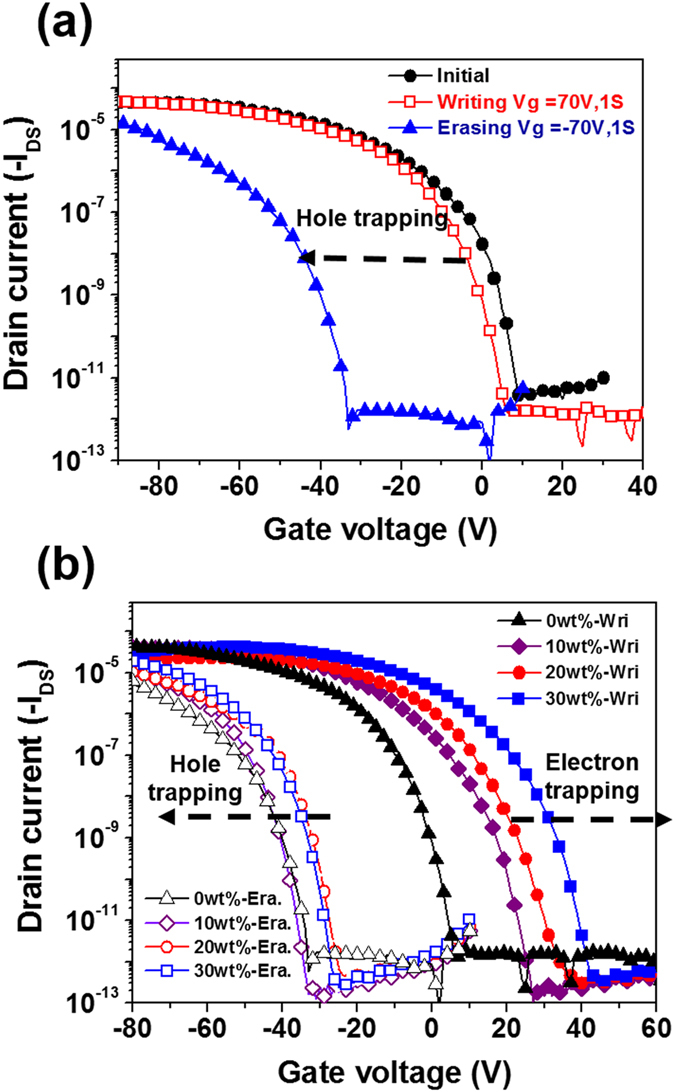
Transfer curves of the fabricated transistor memory devices with (**a**) PVPK and (**b**) the overlapped curves for the writing and erasing processes with ZnOPVPK10, ZnOPVPK20 and ZnOPVPK30. The drain current was measured at *V*_ds_ = −50 V.

To increase the memory window of the ZnO-based floating-gate memory, the hole-trapping PVPK was used as the polymer matrix^38^. The transfer characteristics of the OFET memory devices based on ZnO/PVPK are shown in Fig. 4, the mobilities, the ON/OFF ratio (*I*_on_/*I*_off_), and the V_th_ shifts are also summarized in Table 1. The film with different ZnO composition (0, 10, 20 and 30 wt%), denoted as PVPK, ZnOPVPK10, ZnOPVPK20, ZnOPVPK30. Figure 4a shows the electric characteristics of the device using PVPK and only the negative shift is observed. Figure 4b shows the ZnO/PVPK devices with the “electric bistability” behavior. Compared to that using PVPK (−4.50 V), the *V*_th_ of the nanocomposite after the writing process is shifted to a more positive direction with the values of 11.76 (ZnOPVPK10), 17.54 (ZnOPVPK20) and 21.99V (ZnOPVPK30). Compared to the writing process, the onset position of the erasing curves have inversed trend (−44.48 V for ZnOPVPK10), (−40.42 V for ZnOPVPK20) and (−37.27 V for ZnOPVPK30), as shown in Fig. 4b. The memory window can be improved over 40% from 36.66 V for PVPK to 56.24 V for 10 wt% ZnO. This suggests that the addition of ZnO NPs into PVPK could effectively enhance the electron trapping due to its strong-acceptor behavior. Figure S4 shows the drain current *I*_d_ measured at V_*ds*_ = − 50 V and V_*g*_ = 0 V after the application of different time of gate pulses from 10^−4^ to 10^1^ s under each writing (at V_*ds*_ = 0 V, V_*g*_ = 70 V) and erasing (V_*ds*_ = 0 V, V_*g*_ = −70 V) conditions. The drain current gradually increased from the off-state by increasing the pulse time with a proper gate bias of V_*g*_ = 70 V during programming operation. A similar behavior was observed for the erased drain current. This indicates that at least 0.01 s switching time are typically required to fully switch-on or switch-off the memory devices. In brief, the transparent organic memory devices could be programmed/erased relatively well, but the longer bias pulses (>0.1 s) typically had to be applied to obtain larger V_*th*_ shifts. The switching time is critically depending on the accumulated charge density induced by the applied gate. Therefore, the switching time could be faster by applying higher gate pulses or by reducing the film thickness of the ZnO-polymer nanocomposite. In addition to the writing/erasing efficiency, the photo stability is another important issue for the transparent devices. The device of ZnOPVPK30 showed negligible voltage shifts under an illumination with different wave lengths in the visible light range, as shown in the following Figure S5, indicating good photo stability. The photo-stable performance may be attributed to low absorbance in the visible light range. Additionally, the very thin pentacene layer (30 nm) may be one of reasons for low photosensitivity.

**Figure 5:**
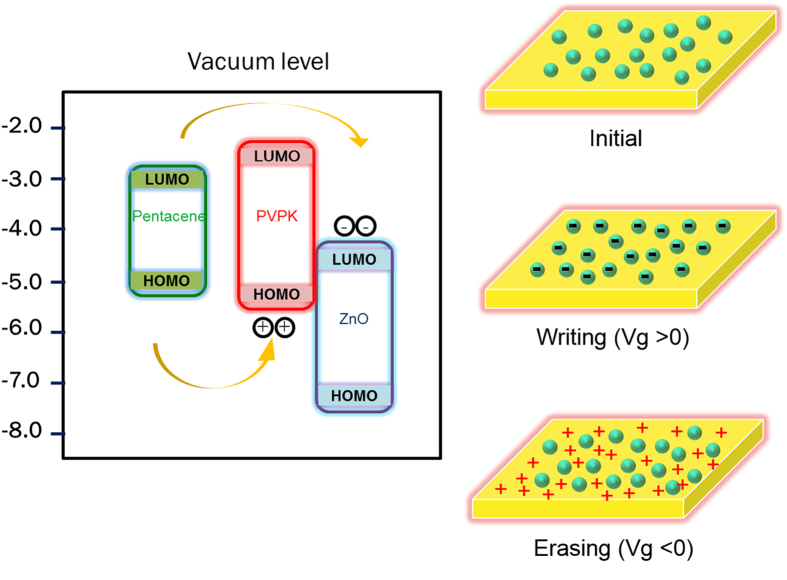
Proposed mechanisms and energy band diagrams under positive and negative bias for pentacene OFET memory using PVPK/ZnO as electrets.

Here we proposed a possible mechanism in terms of the energy level diagram for ZnO/PVPK in Fig. 5. The charge transfer from pentacene into the charge storage sites is induced by the applied gate electric field. Due to the external electrical field, the electrons on the LUMO energy level of pentacene may overcome the energy barrier of the PVPK matrix and are transferred to ZnO. The lower LUMO level (−4.4 eV) of ZnO than that of pentacene (−2.9 eV) could stabilize the trapped electrons and thus the trapped electron charges would not be transferred back to pentacene without external voltage bias. It is energetically favorable for charge transfer when blending a strong acceptor into the dielectric. The electrons retained in ZnO change the built-in electric field under the applied electric field, and thus the hole carriers of pentacene are easily accumulated at the interface, leading to a profound positive *V*_th_ shift. For the erasing process, the hole carrier in the channel would inject and neutralized the electrons in PVPK/ZnO, leading to returning to the OFF state of the device. In the ZnO/PS composite, ZnO plays the role of an electron acceptor in the storage layer. However, in the ZnO/PVPK system, the stored electrons might not be fully detrapped in the erasing process due to the hole transporting characteristic of PVPK, leading to the recombination between the electrons and holes. The residue electrons in the dielectric after the erasing process would become more in the higher ZnO composition. Thus, we found a slightly smaller *V*_*th*_ shift with the increasing amount of ZnO loading in ZnO/PVPK system compared to the pure PVPK system.

### The Retention Characteristics

**Figure 6:**
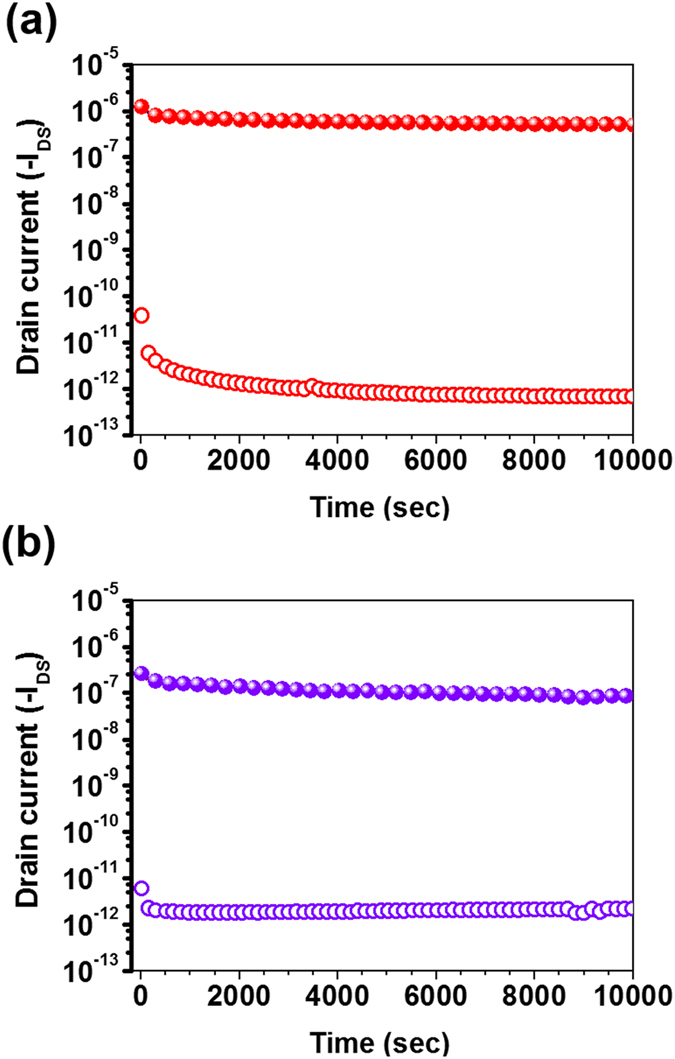
Retention times of the pentacene-based OFET memory devices with (**a**) ZnOPS30 and (**b**) ZnOPVPK30 dielectrics were measured at a drain–source of −50 V.

**Figure 7:**
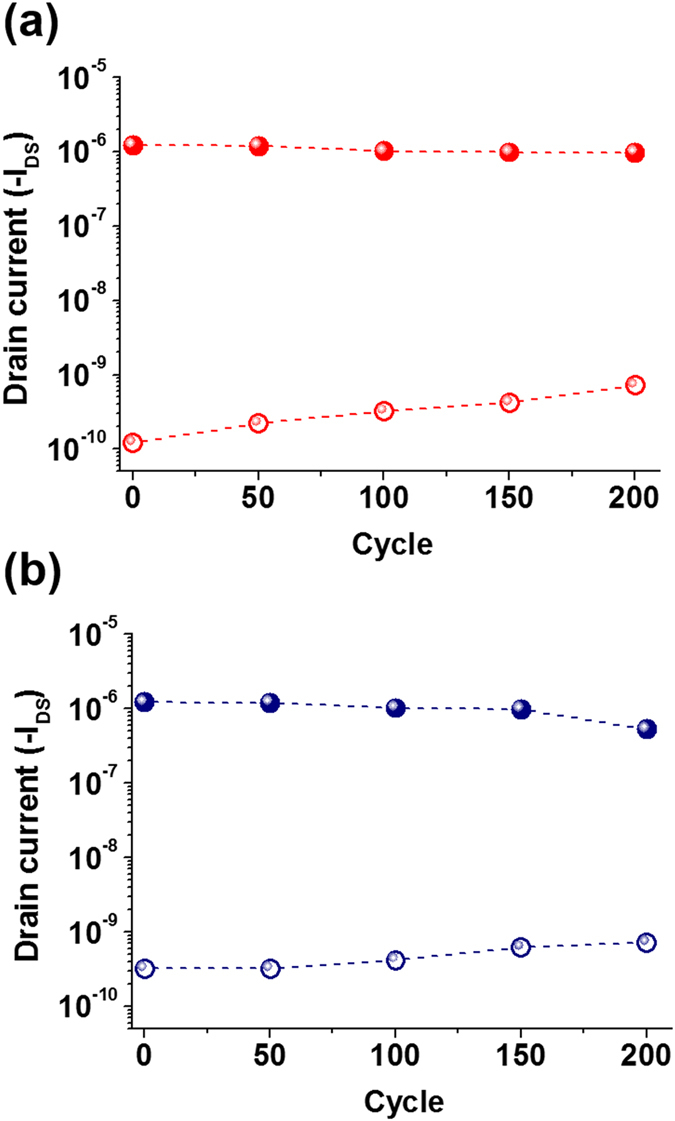
Reversible switching for the ON and OFF states for the device of the pentacene-based OFET memory devices with (**a**) ZnOPS30 and (**b**) ZnOPVPK30.

Long-term data retention properties are essential for nonvolatile memory applications. The charge retention characteristics of the transparent OFET memory devices are shown in Fig. 6. The re**t**ention capability of the OFET memory devices using the ZnO/PS and ZnO/PVPK electrets was measured after the application of + 70 V (ON state) and −70 V (OFF state) gate pulses for 1 s. To compare the endurance of the behavior in different composites (ZnO-PS and ZnO-PVPK), we evaluated the charge retention time in the devices using the same ZnO composition of ZnOPS30 and ZnOPVPK30. Similar experiment result has been observed for both of ZnO-PS and ZnO-PVPK systems. When reading at a gate-source voltage of −10 V, the high and low conducting states can keep over 10^5^ s, and high ON/OFF current ratio (>10^6^) is obtained, implying no significant charge leakage. The high ON/OFF ratio make “0” and “1” clearly be distinguished in these memory devices. This could be contributed to the good dispersion of ZnO in polymer dielectric, which could prevent lateral charge diffusion, the slow relaxation of charges renders the superior operating reliability of the device. To evaluate the operating stability of the electrical switching, the WRER (writing/reading/erasing/reading) cycles were performed and shown in Fig. 7. Note that the writing, reading and erasing voltages are set to the *V*_*g*_ of 70, −10 and −70 V, respectively. The responding ON and OFF currents of ZnOPS30 are maintained over 200 cycles with the on/off current ratio of ca. 1.3 × 10^3^, showing an excellent stress endurance, which reveals the promising potential applications for nonvolatile transistor memory devices. This may be attributed to that the insulating PS matrix incorporated with the separated ZnO NPs provides a high energy barrier between ZnO and pentacene to effectively suppresses the occurrence of current leakage during the readout. In ZnOPVPK30, the OFF current is relatively stable to the ZnO/PS system because of the hole transporting property of dielectric, PVPK. The on/off current ratio shows a small decrease from 3.7 × 10^3^ to 7.28 × 10^2^. This difference is possibly attributed to the recombination between the electrons and holes. During the repeated writing and erasing cycling, the stored holes in the PVPK matrix might recombine the electrons trapped in ZnO, thus resulting in decreasing the amount of trapped electrons during the writing process.

### Conclusion

Transparent nonvolatile nano-floating gate memory devices are demonstrated through using ZnO NPs/polymer electret nanocomposite as a charge storage layer. The ZnO NPs were treated by the ligand, n-butylamine, leading the stable and well-separated NPs in solution. Furthermore, high transparent dielectric layers can be made, despite adding ZnO NPs into PS or PVPK polymer electret. Spray-coated PEDOT:PSS was exploited as the gate and source–drain electrodes for the fabrication of the transparent memory devices. The overall transmittance is 90% at the wavelength of 500 nm, showing high potential for transparent memory. A large memory window, long charge retention time over 10^5^ s and the WRER process over 200 cycles were obtained in this ZnO nanocomposite-based memory devices. Additionally, all of the processes were carried out in the low temperature and without using noble metals as floating-gate. Therefore, these methods could potentially be used in integrated electronic components/circuits and transparent electronic device applications.

## Methods

### Materials

PEDOT:PSS solutions (CLEVIOS PH 1000) were purchased from Heraeus. The solid content of the PH 1000 solution was 1–1.3% and had a PEDOT to PSS ratio of 1:2.5 by weight. Poly(4-vinylphenol) (PVP,MW = 20 000 g mol^−1^), polystyrene (PS,MW = g mol^−1^), Zonyl FS-300 (Zonyl) and DMSO were purchased from Aldrich and used without further treatment. The poly 9-(4-vinylphenyl) carbazole (PVPK) were synthesized according to the literature method.

### ZnO NPs Synthesis

ZnO NPs were prepared using an adapted procedure based on the work of Weller. The general procedure used for the preparation of NPs was as follows: zinc acetate dihydrate (Acros, >98%, 2.95 g, 13.4 mmol) was dissolved in methanol (125 mL) at 60 °C, a solution of KOH (Merck, 87%, 1.48 g, 23 mmol) in methanol (65 mL) was added in 10 min to the zinc acetate dihydrate solution under vigorous stirring. Zinc hydroxides precipitated but dissolved again. After 5 min, the solution became translucent and remained translucent. After 1.5 h, the NPs started to precipitate and the solution became turbid. Precipitate and mother liquor were separated, and the precipitate was washed twice with methanol (50 mL). After the washing steps (5 min), the suspension was left unstirred for a minimum of 1 h to reach full precipitation. The washed precipitate was treated with n-butylamine and dissolving by the mixing solvent of methanol: chloroform = 1:3. This solution was only slightly translucent, almost transparent, and was stable for more than two weeks.

### Transistor Memory Device fabrication

The schematic configuration of the fabricated pentacene based transistor memory devices using ZnO/PVPK as nano floating-gate is shown in Fig. 2b. The memory devices were fabricated on an ITO glass and PEDOT:PSS was used for transparent electrode. Cross-linked PVP acting as a blocking organic dielectric was prepared from 12 wt% PVP and 5 wt% cross-linking agent (poly(melamine-coformaldehyde, PMF) in propylene glycol monomethyl ether acetate (PGMEA). The PVP solutions were filtered through a 0.22 μm polytetrafluoroethylene (PTFE) syringe filter. The crosslinked PVP blocking organic dielectric layer was spin-coated and then cured at 180 °C for 2 h in a vacuum oven. The polymer/ZnO NPs solutions were prepared in chloroform solution (10 mg/ml) and were stirred overnight to obtain homogeneous dispersion. Thereafter, the solution was filtered through the PTFE membrane syringe filter (pore size, 0.22 μm) and then spin-coated onto the cPVP layer at 1000 rpm for 60 s. The thickness of the cross-linked PVP, PS and PVPK are 400, 55 and 50 nm, respectively, which was measured using Micro figure Measuring Instrument. The PEDOT:PSS solution containing 5% DMSO was filtered through a syringe filter (0.22 μm pore size) to remove precipitated material for the ink of spray coating. Prior to the deposition of the PEDOT:PSS , 1 wt% Zonyl was added to the PEDOT:PSS solution to enhance the wettability of PEDOT:PSS on hydrophobic substrate. Where noted in the main text, 1% Zonyl by volume was added to the PEDOT:PSS. Shadow mask was used to pattern source-drain, and the channel length (L) and width (W) of the devices were 100 and 2000 μm, respectively. Figure S6 shows the transparency testing of the PEDOT:PSS electrodes with different thickness. The sheet resistance is about 1200(Ω/sq) at a film thickness of 298 nm. The pentacene was thermally deposited with a deposition rate of 0.35 nm s^−1^ at 90 °C under vacuum (10^−7^ torr) to form the 30 nm thick film.

### Characterization

The degree of aggregation of NPs were performed through field emission scanning electron microscope (FE-SEM, JEOL JSM-6330F). The energy level of the polymers were investigated by cyclic voltammetry (CV)^39^ and the thickness of polymer film was measured with Microfigure Measuring Instrument (Surfcorder ET3000, Kosaka Laboratory Ltd.).The morphology of polymer thin film surface were obtained with a Nanoscope 3D Controller atomic force micrographs (AFM, Digital Instruments) operated in the tapping mode at room temperature. The electrical characteristics of the fabricated transistor memory devices were measured using a Keithley 4200 semiconductor parametric analyzer. The field-effect mobility *μ*_FET_ (cm^2^V^−1^s^−1^), on/off ratios (I_on_/_off_), and threshold voltages (*V*_th_) of these fabricated devices were obtained using equation (1) in the saturation regime:


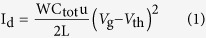


where I_d_ is the drain current, *V*_g_ is the gate voltage, *V*_th_ is the threshold voltage, μ is the hole mobility, W is the channel width, L is the channel length, and *C*_tot_ is the capacitance per unit area of total dielectric layer, respectively.

## Additional Information

**How to cite this article**: Shih, C.-C. *et al.* High Performance Transparent Transistor Memory Devices Using Nano-Floating Gate of Polymer/ZnO Nanocomposites. *Sci. Rep.* **6**, 20129; doi: 10.1038/srep20129 (2016).

## Supplementary Material

Supplementary Information

## Figures and Tables

**Table 1:** The electrical and memory performance of OFET memory device with ZnO NPs blended with PS and PVPK as charge-storage layer.

	*μ*_ave_ [cm^2^V^−1^s^−1^]	*I*_ON_/*I*_OFF_	*V*_th, ave_ [V]	*V*_th, ave_ [V]		Memory window [V]
				Writing process	Erasing process	
PS	0.32 ± 0.02	10^6^	−5.90 ± 0.8	−7.50 ± 0.5	−9.20 ± 0.3	4.23
ZnOPS10	0.36 ± 0.01	10^6^	−8.87 ± 0.5	12.27 ± 1.2	−10.87 ± 1.3	23.14
ZnOPS20	0.58 ± 0.03	10^6^	−9.29 ± 0.2	17.54 ± 2.1	−10.21 ± 1.2	27.75
ZnOPS30	0.62 ± 0.05	10^5^	−8.53 ± 0.7	21.99 ± 1.2	−9.27 ± 1.1	31.26
PVPK	0.12 ± 0.02	10^6^	−4.50 ± 0.8	−7.50 ± 0.3	−47.50 ± 1.2	36.55
ZnOPVPK10	0.36 ± 0.01	10^6^	−9.42 ± 0.5	11.76 ± 1.2	−44.48 ± 1.7	56.24
ZnOPVPK20	0.40 ± 0.03	10^6^	−9.78 ± 0.2	18.54 ± 2.4	−40.42 ± 2.3	58.96
ZnOPVPK30	0.48 ± 0.05	10^6^	−10.2 ± 0.7	22.98 ± 1.4	−37.27 ± 0.8	60.25
