# Supplementary material for: High Performance Transparent Transistor Memory Devices Using Nano-Floating Gate of Polymer/ZnO Nanocomposites
Source: Sci Rep. 2016 Feb 1;6:20129. doi: 10.1038/srep20129 (PMC4735596; doi:10.1038/srep20129)
Supplement: Supplementary Information [file srep20129-s1.doc]

Electronic Supplementary Information

**High Performance Transparent Transistor Memory Devices Using Nano-Floating Gate of Polymer/ZnO Nanocomposites**

*Chien-Chung Shih,^1^Wen-Ya Lee,**^2^ Yu-Chen Chiu,^1^Han-Wen Hsu,^3^Hsuan-Chun Chang,^1^ Cheng-Liang Liu,^3^ and Wen-Chang Chen^1^**

^1^Department of Chemical Engineering, National Taiwan University, Taipei, 10617 Taiwan E-mail: chenwc@ntu.edu.tw

^2^ Department of Chemical Engineering and Biotechnology, National Taipei University of Technology, Taipei, 10608
^3^Department of Chemical and Materials Engineering, National Central University, Taoyuan 32001, Taiwan.

Tel: 886-2-23628398, Fax: 886-2-23623040

E-mail:chenwc@ntu.edu.tw

**
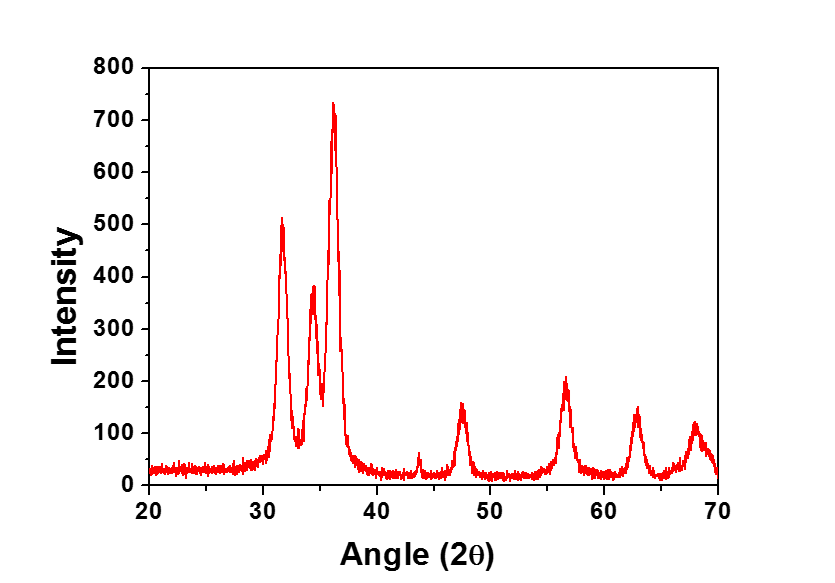
**

**Figure S1.** (a) X-ray pattern of the powder of the ZnO nanoparticles.

**
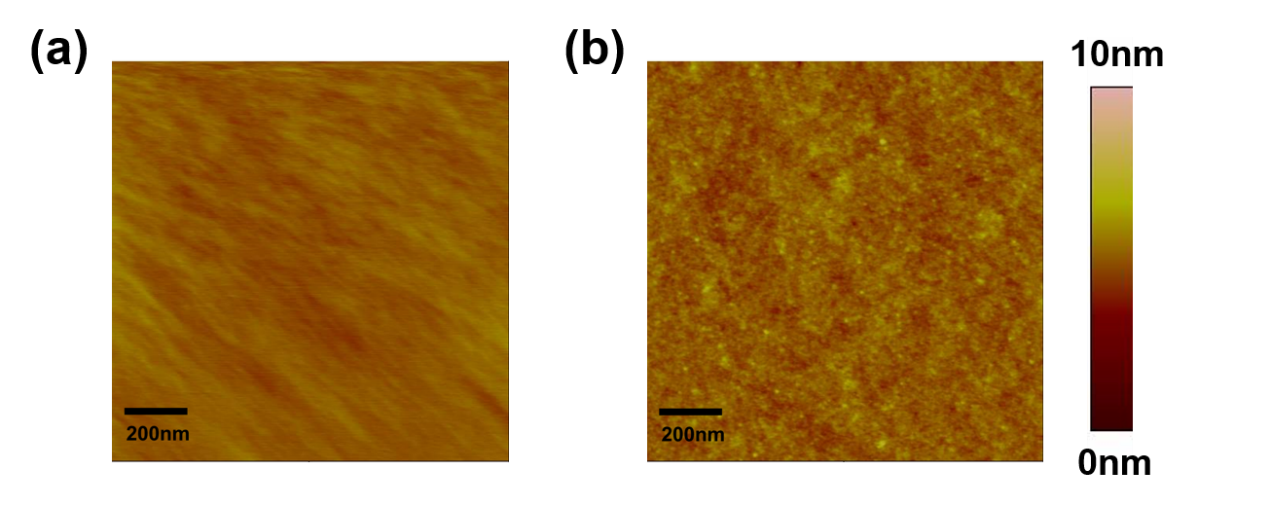
**

**Figure S2.** AFM topographies of thin films (a)ZnOPS-30 and (b)ZnO/PVPK-30


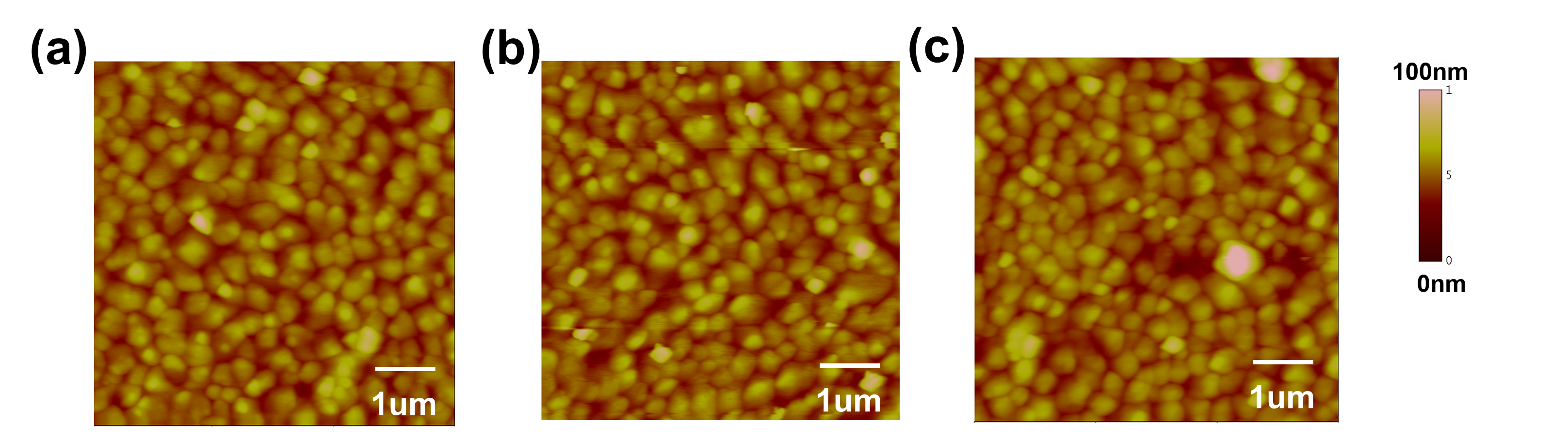


**Figure S3.** AFM topographies of pentacene deposited on dielectric layer (a)ZnOPS-10 and (b)ZnO/PVPK-20 (c)ZnOPS-30

**
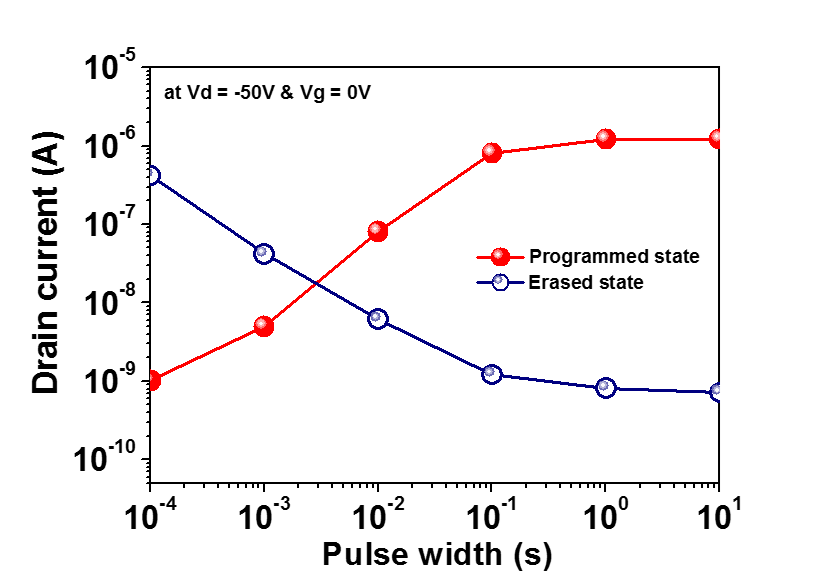
**

**Figure S4** Change in drain current of memory transistors as function of pulse width. Devices were programmed/erased by applying gate pulse voltages of +70 and -70 V, respectively.

**
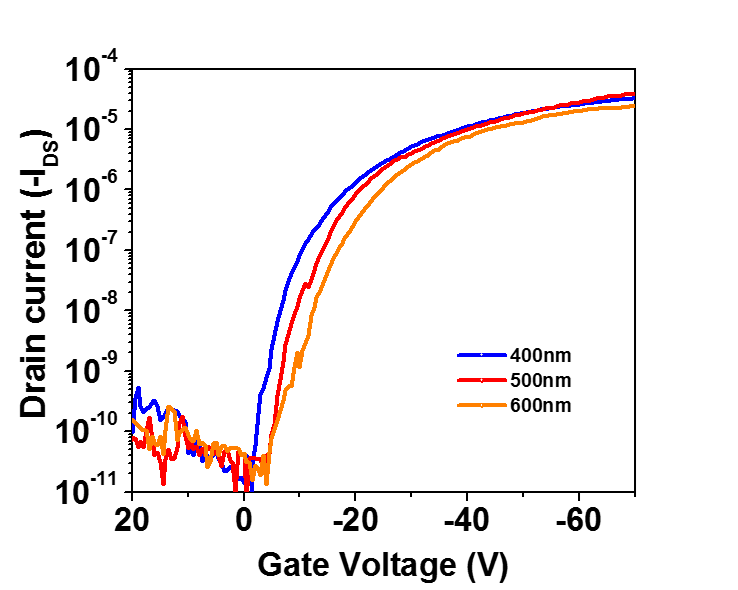
**

**Figure S5.** The device of ZnOPVPK30 under an illumination with different wave lengths in the visible light range

**
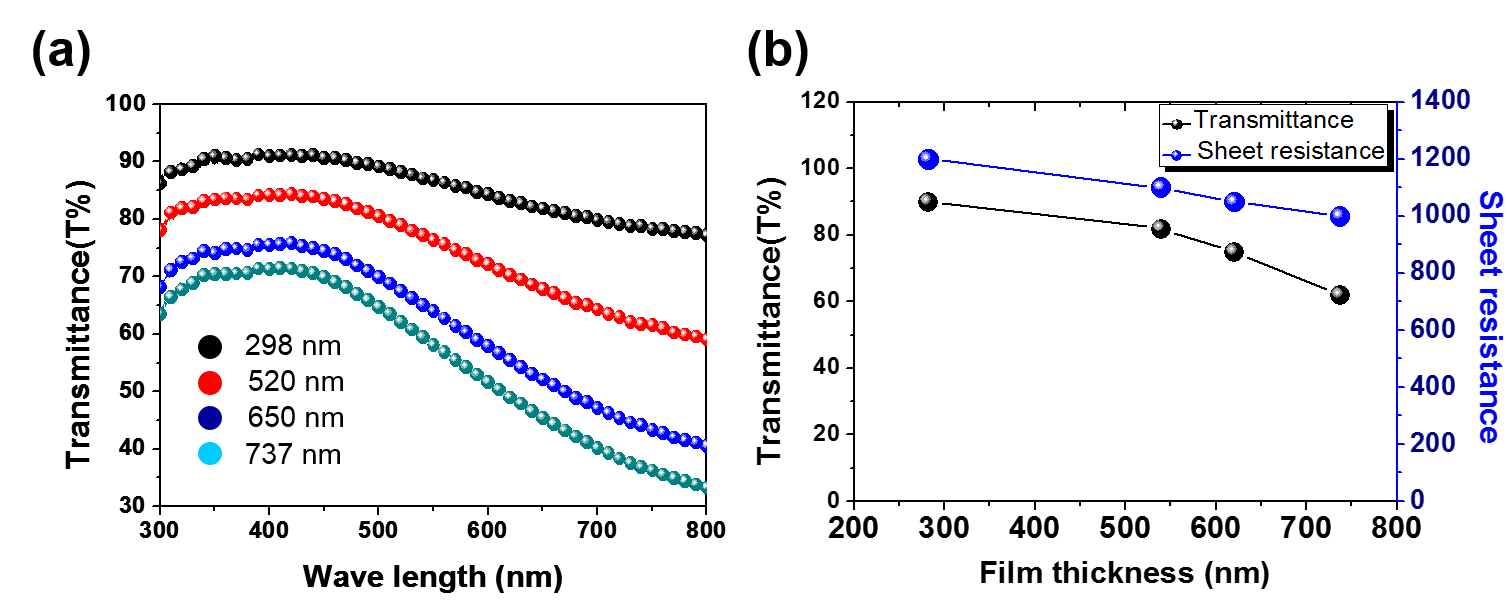
**

**Figure S6.** Spray coating of PEDOT:PSS electrode with different film thickness and their relationships with transmittance and sheet resistance
